# Relationship between the Composition of Flavonoids and Flower Colors Variation in Tropical Water Lily (*Nymphaea*) Cultivars

**DOI:** 10.1371/journal.pone.0034335

**Published:** 2012-04-02

**Authors:** Manlan Zhu, Xuchen Zheng, Qingyan Shu, Hui Li, Peixing Zhong, Huijin Zhang, Yanjun Xu, Lijin Wang, Liangsheng Wang

**Affiliations:** 1 Beijing Botanical Garden/ The Key Laboratory of Plant Resources, Institute of Botany, The Chinese Academy of Sciences, Beijing, China; 2 College of Horticulture, Nanjing Agricultural University, Nanjing, China; 3 Graduate University of the Chinese Academy of Sciences, Beijing, China; 4 Department of Applied Chemistry, China Agricultural University, Beijing, China; University of Georgia, United States of America

## Abstract

Water lily, the member of the Nymphaeaceae family, is the symbol of Buddhism and Brahmanism in India. Despite its limited researches on flower color variations and formation mechanism, water lily has background of blue flowers and displays an exceptionally wide diversity of flower colors from purple, red, blue to yellow, in nature. In this study, 34 flavonoids were identified among 35 tropical cultivars by high-performance liquid chromatography (HPLC) with photodiode array detection (DAD) and electrospray ionization mass spectrometry (ESI-MS). Among them, four anthocyanins: delphinidin 3-*O*-rhamnosyl-5-*O*-galactoside (Dp3Rh5Ga), delphinidin 3-*O*-(2″-*O*-galloyl-6″-*O*-oxalyl-rhamnoside) (Dp3galloyl-oxalylRh), delphinidin 3-*O*-(6″-*O*-acetyl-β-glucopyranoside) (Dp3acetylG) and cyanidin 3- *O*-(2″-*O*-galloyl-galactopyranoside)-5-*O*-rhamnoside (Cy3galloylGa5Rh), one chalcone: chalcononaringenin 2′-*O*-galactoside (Chal2′Ga) and twelve flavonols: myricetin 7-*O*-rhamnosyl-(1→2)-rhamnoside (My7RhRh), quercetin 7-*O*-galactosyl-(1→2)-rhamnoside (Qu7GaRh), quercetin 7-*O*-galactoside (Qu7Ga), kaempferol 7-*O*-galactosyl-(1→2)-rhamnoside (Km7GaRh), myricetin 3-*O*-galactoside (My3Ga), kaempferol 7-*O*-galloylgalactosyl-(1→2)-rhamnoside (Km7galloylGaRh), myricetin 3-*O*-galloylrhamnoside (My3galloylRh), kaempferol 3-*O*-galactoside (Km3Ga), isorhamnetin 7-*O*-galactoside (Is7Ga), isorhamnetin 7-*O*-xyloside (Is7Xy), kaempferol 3-*O*-(3″-acetylrhamnoside) (Km3-3″acetylRh) and quercetin 3-*O*-acetylgalactoside (Qu3acetylGa) were identified in the petals of tropic water lily for the first time. Meanwhile a multivariate analysis was used to explore the relationship between pigments and flower color. By comparing, the cultivars which were detected delphinidin 3-galactoside (Dp3Ga) presented amaranth, and detected delphinidin 3′-galactoside (Dp3′Ga) presented blue. However, the derivatives of delphinidin and cyanidin were more complicated in red group. No anthocyanins were detected within white and yellow group. At the same time a possible flavonoid biosynthesis pathway of tropical water lily was presumed putatively. These studies will help to elucidate the evolution mechanism on the formation of flower colors and provide theoretical basis for outcross breeding and developing health care products from this plant.

## Introduction

**Figure 1:**
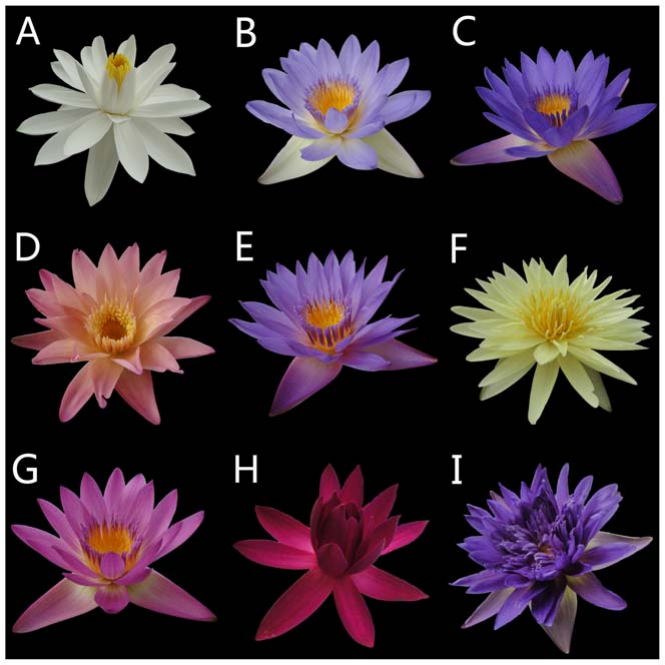
Nine representative flower colors, ‘Ai Ji Bai’ (A), 18 (B), 27 (C), ‘Albert Greenberg’ (D), 34 (E), ‘Eldorado’ (F), 37 (G), ‘Roxburgh’ (H), and ‘Tai Guo Wang’ (I), for water lily cultivars sampled from a natural population.

Water lily (an aquatic herb of genus *Nymphaea*, family Nymphaeaceae), a precious perennial aquatic flower plant, is divided into two ecological groups, namely Tropical and Hardy water lily [1]. There are about 50 species in the whole world, five of which originate from China: *N. alba* L., *N. candida* Presl., *N. tetragona* Georgi., *N. lotus* L.var. *pubescens* and *N. atellata* Willd [2]. It is called subaqueous nymph and symbolized as spotlessness, trueness and coquettishness. Like lotus, water lily is not only an ornamental plant but also an important water purification one. Because the roots of water lily can absorb the poisonous substances like mercury, lead, phenol, etc and filter the microorganism in water, it plays an important role in decontaminating water, afforesting and landscaping [3], [4]. In the meanwhile, flowers and roots of water lily can both be made into tea and liquor, and the whole plant has been useful in the therapies of nephritis and is reputedly a detoxicant and aphrodisiac along with astringent, diuretic properties [5]. Furthermore, as shown in Fig. 1, tropical water lily owns flowers with the special colors of blue, violet and bluish purple which hardy water lily lacks of, for this reason the former is more favorable by people. However, little is known about the formation and genetic mechanism of the flower colors on tropical water lily, the study on its pigments of flower petals will illuminate the formation of flower colors.

Flavonoid is the decisive pigment presented in most flower colors, among which anthocyanin is the key component. Flower petals with anthocyanin present red, pink, purple and blue. Except contribution of flower color, it also has important biological activities, such as antioxidative, antiinflammatory, antimicrobial, anti-platelet aggregation and antitumor activity, etc. [6]–[9]. At present, TLC (Thin-Layer Chromatography), HPLC-DAD, HPLC-MS^n^ (multi-stage tandem Mass Spectrometry), UV-vis (Ultraviolet visible), HSCCC (High Speed Countercurrent Chromatography) and NMR (Nuclear Magnetic Resonance) are the important techniques in characterizing the distribution and identifying the structure of anthocyanins and have been used in many plants, like tree peony [10]–[12], purple corn [13], *Ficus carica* L. [14] and *Vaccinium myrtillus* [15].

Till now, there is no systematic study on the pigment constitutes of water lily petals except several limited reports by Fossen et al.. Among those reports, 9 glycosides of anthocyanidin were isolated from the red flowers and leaves of *Nymphaba*×*marliacea* var. *Escarboucle*, reddish leaves of *N. alba* and blue flowers of *N. caerulea* [16]–[19], and 11 glycosides of flavonol from the red petals and leaves of *Nymphaba*×*marliacea* var. *Escarboucle* and blue flowers of *N. caerulea* by a combination of chromatography, homo- and heteronuclear two-dimensional NMR techniques and electrospray MS [20], [21]. Apart from those literatures, other articles only concentrated on one species or one cultivar [22]–[26]. Due to the limitation of techniques and the limited number of accession, the real quantity and type of flavonoids presented in the flower petals of water lily remains unclear. It is necessary to use as many as possible accessions to characterize the distribution and identify the structure of anthocyanins in water lily plants, in order to provide a global knowledge on the flower color formation. Meantime, it will provide a theoretical basis for selection parents of breeding novel cultivars with optimal flower colors by outcrossing. In this study, chromatographic conditions were optimized in order to obtain higher separation efficiency and peak resolution of target compounds, and at the same time a rapid method by HPLC-DAD coupled with ESI-MS was established to detect anthocyanins and other flavonoids simultaneously and to analysis those compounds qualitatively and semi-quantitatively. The relationship between flower color and pigment composition was also discussed. The established technique will be helpful to obtain fingerprints for these plants, and isolate the important components for medical therapy or study on its anti-oxidant ability, parental selection for outcrossing and breeding. It is also important to explore the rare blue coloration of this plant, which will be a base for the breeding new cultivars with special colors within Nymphaeaceae family. The components of flower pigments will also be an important data for classification of cultivars.

## Results

### Identification of Flavonoids

In order to obtain higher separation efficiency and peak resolution of target compounds, chromatographic conditions were optimized (Figure S1). The developed method provided satisfactory precision and accuracy with over-all intra-day and inter-day variations of 0.03%–0.75% and 0.03%–3.5%, respectively (Table S1). All calibration curves showed good linear regression (r^2^≥0.9986) within test ranges. The limit of detection (LOD) of optimized method was 0.4537 and 0.7193 µg/mL for MV3G5G and rutin, respectively, while the limit of quantification (LOQ) was 1.5124 and 2.3977 µg/mL (Table S2).

Generally, glycosylation sites usually occurs at the 7-hydroxyl group for flavones and flavanones, the 3- and 7-hydroxyl for flavonols and flavan-3-ols, and the 3- and 5-hydroxyl for anthocyanidins [27]. Sugars combined with the aglycone are always hexose and pentose. Glucose is the most commonly encountered sugar, galactose, rhamnose, xylose and arabinose are not uncommon. But in the cultivars of water lily, galactose is more common one instead of glucose [16]–[21]. Acylated glycosides, in which one or more of the sugar hydroxyls are esterified with an acid, also occur. There are many kinds of acids which usually participate in acylation like acetic, oxalic, gallic, cinnamic, ferulic acid and so on [18], [19]. In this research the structure of flavonoids were deduced mostly through retention time of the HPLC analysis, elution order, UV-vis spectroscopy and MS^n^, and by comparing with the standard and the known structures published in other researchers, 11 anthocyanins and 22 glycosides of flavonol as well as one chalcone were detected and in the meanwhile the structure of them were identified or identified tentatively (Table 1 and Table S3).

**Table 1:** HPLC-DAD and HPLC-ESI-MS analysis of anthocyanins in water lily petals as well as the characterization and tentative identification.

NO^1^.	Identifacation/tentative identification	tR (min)	λ_max_ (nm)	E440/Evis-max (%)	EST-(+)-MS (*m/z*)	References
**a1**	delphinidin 3-*O*-rhamnosyl-5-*O*-galactoside	9.88	267,525	26.6	611[M]^+^,465,449,303	
a2	delphinidin 3′-*O*-(2″-*O*-galloyl-β-galactopyranoside)	9.90	274,515	39.2	617[M]^+^,455,303	Fossen Torgils, Andersenet Øyvind M.,1999
a3	delphinidin 3-*O*-(2″-*O*-galloyl-β-galactopyranoside)	10.47	278,527	24.5	617[M]^+^,455,303	Fossen Torgils, Andersenet Øyvind M.,1999Fossen Torgils et al.,1998Fossen Torgils et al.,1997
**a4**	delphinidin 3-*O*-(2″-*O*-galloyl-6″-*O*-oxalyl-rhamnoside)	11.51	278,529	23.7	673[M]^+^,601,449,303	
a5	cyanidin derivatives	13.38	280,519	28.2	287	
**a6**	cyanidin 3- *O*-(2″-*O*-galloyl-galactopyranoside)-5-*O*-rhamnoside	14.53	281,519	27.9	769[M+Na]^+^,747[M]^+^,601,449,287	
a7	delphinidin 3′-*O*-(2″-*O*-galloyl-6″-*O*-acetyl-β-galactopyranoside)	16.41	278,517	38.6	659[M]^+^,455,303	Fossen Torgils, Andersenet Øyvind M.,1999
**a8**	delphinidin 3-*O*-(6″-*O*-acetyl-β-glucopyranoside)	16.65	278,527	27.0	507[M]^+^,465,303	
a9	delphinidin 3-*O*-(2″-*O*-galloyl-6″-*O*-acetyl-β-galactopyranoside)	18.37	279,529	23.4	659[M]^+^,465,455,303	Fossen Torgils, Andersenet Øyvind M.,1999Fossen Torgils et al.,1998Fossen Torgils et al.,1997
a10	unknow	20.20	278,510	46.3		
a11	cyanidin 3-*O*-(2″-*O*-galloyl-6″-*O*-acetyl-β-galactopyranoside)	21.69	280,521	27.5	643[M]^+^,481,287	Fossen Torgils et al.,1998

1 : The bold numbers of compounds were reported for the first time in tropic water lily.

#### Qualitative analysis for anthocyanins

**Figure 2:**
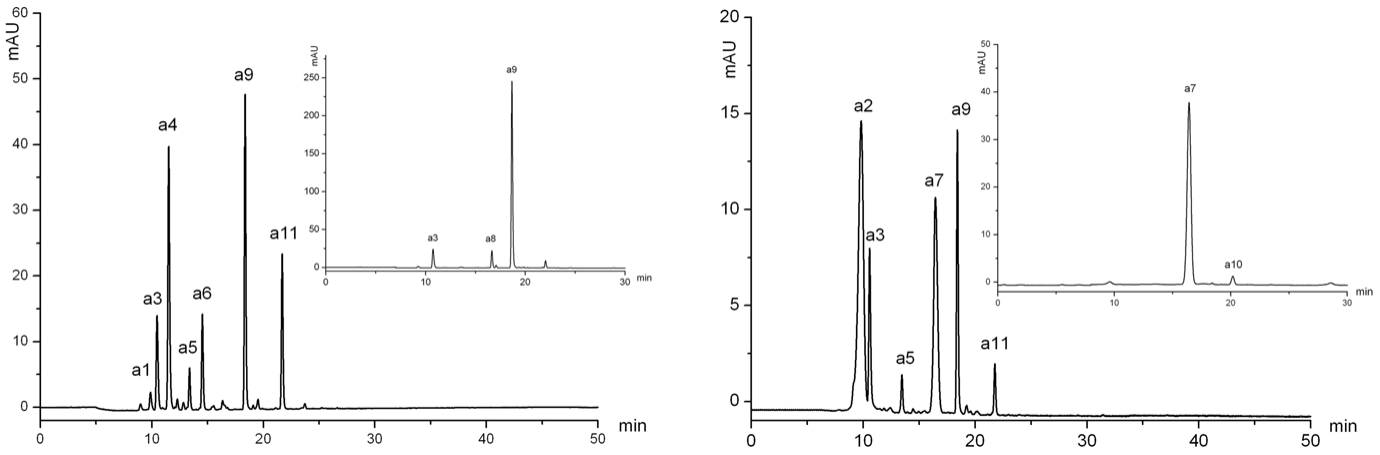
The anthocyanin HPLC profiles of water lily petals (detected at 525 nm).

**Figure 3:**
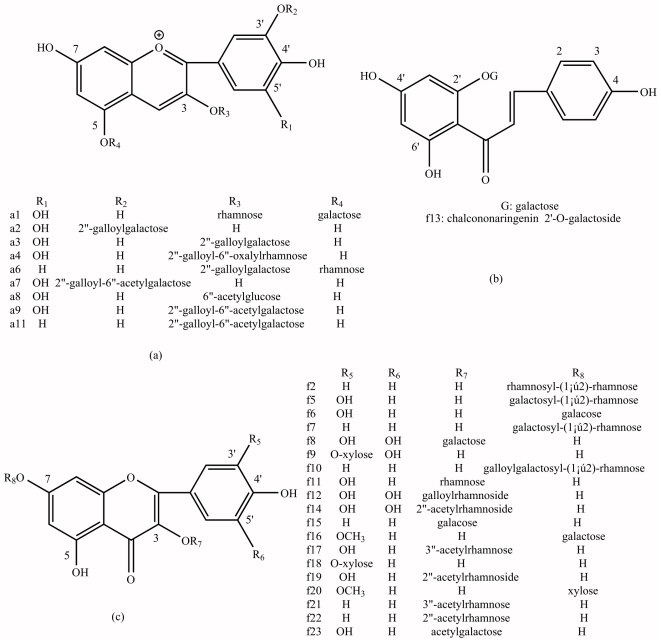
The chemical structure scheme of flavonoids detected in petals of water lily.

The HPLC chromatogram detected the extract aqua in the visible region at 525 nm, showed 11 anthocyanins, **a1**–**a11** (Fig. 2) (the chemical structure demonstrated in Fig. 3). It showed that there were only two aglycones, delphinidin and cyanidin in the petals of water lily. Among those anthocyanins, 5 compounds (**a2**, **a3**, **a7**, **a9** and **a11**) were already reported in the past researches. By the retention time of the HPLC analysis, elution order and UV-vis spectroscopy (Table 1), they were identified to be delphinidin 3′-*O*-(2″-*O*-galloyl-β-galactopyranoside) (Dp3′galloylGa) (**a2**), delphinidin 3-*O*-(2″-*O*-galloyl-β-galactopyranoside) (Dp3galloylGa) (**a3**), delphinidin 3′-*O*-(2″-O-galloyl-6″-*O*-acetyl-β-galactopyranoside) (Dp3′galloy-acetylGa) (**a7**), delphinidin 3-*O*-(2″-*O*-galloyl-6″-*O*-acetyl-β-galactopyranoside) (Dp3galloyl-acetylGa) (**a9**) and cyanidin 3-*O*-(2″-*O*-galloyl-6″-*O*-acetyl-β-galactopyranoside) (Cy3galloyl-acetylGa) (**a11**), and were verified by electrospray MS. In the rest of the compounds, **a1**, **a4** and **a8** have fragment ions at *m/z* 303 which corresponding to delphinidin aglycone, so these two components are presumed to be delphinidin derivatives. **a5** and **a6** have fragment ions at *m/z* 287 which corresponding to cyanidin aglycone, so they are presumed to be cyanidin derivatives. Owing to the low amount in samples and little information of MS, **a10** could not been identified.

From Table 1, the value of E_440_/E_vis-max_ (the ratio of absorbance value at 440 nm and that at visible maximum absorption wavelength) [28] about 11 anthocyanins is between 23%–47%. Glycosylation at different position discriminated the value of E_440_/E_vis-max_. We can see that when the glycosylation is at 3′-OH, the value is about 39% higher than that of at 3-OH, meanwhile the two delphinidin 3′-glycosides, **a2** and **a7**, showed a hypsochromic shift at 12 nm in the UV-vis spectrum compared with the analogous of delphinidin 3-glycosides, **a3** and **a9**. Fossen et al. have already reported the identification of some anthocyanins which acylated with acetic and (or) gallic acid, glycosylated with monosaccharide galactose instead of glucose, and the acetyl and galloyl group was determined to be situated in the 6″-position and the 2″-position on the sugar respectively [18], [19].

The MS data of **a1**, fragment ion at *m/z* 465 ([M+H-146u]^+^) and 449 ([M+H-162u]^+^), exhibited two sugars link to delphinidin aglycone, and the relative abundance of *m/z* 449 was higher than that of *m/z* 465 which demonstrated the molecular ion 611 ([M+H]^+^) loses *m/z* 162u easier. Because glycosidic bond at 5-position is cracked easily [29], we identified **a1** as delphinidin 3-*O*-rhamonsyl-5-*O*-galactoside (Dp3Rh5Rh) tentatively. Peak **a4** was deduced as delphinidin glycoside based on the following information: the protonated molecule ion at *m/z* 673 ([M+H]^+^), the Y_0_^+^ at *m/z* 303, and other fragment ions at *m/z* 601 ([M+H-72u]^+^) and 449 ([M+H-(72+152)u]^+^). So the structure of **a4** was assigned as delphinidin 3-*O*-(2″-*O*-galloyl-6″-*O*-oxalyl-rhamnoside) (Dp3galloyl-oxalylRh). Peak **a8** had the molecule ion at *m/z* 507 ([M+H]^+^), fragment ions at *m/z* 465 [M+H-42]^+^ and the Y_0_^+^ at *m/z* 303. It was in line with delphinidin 3-*O*-(6″-*O*-acetyl-β-galactopyranoside) (Dp3acetylGa) which had been reported in the paper of Fossen et al. [17]. So it was likely to be Dp3acetylGa. However, two similar compounds of **a8** had been found in petals of Hardy water lily and the retention time of one compound was close to that of **a8**, the other was a little earlier than it (unpublished data). Because the retention time of glucose was later than that of galactose in HPLC, we tentatively identified **a8** as 3-*O*-(6″-*O*-acetyl-β-glucopyranoside) (Dp3acetylG). The spectra of peak **a6** detected in positive full scan mode showed the sodium adduct at *m/z* 769 ([M+Na]^+^) and the molecule ion at *m/z* 747 ([M+H]^+^) corresponding to the successive losses of sugar units and acyl group, and finally gave the protonated aglycone Y_0_^+^ (*m/z* 287), fragment ions at *m/z* 601 (M+H-146u]^+^), 449 ([M+H-(146+152)]^+^) and 439 ([M+H-(146+162)]^+^). As a result, the relative abundance about *m/z* 449 was higher than that of *m/z* 439. We could conclude that rhamnose was linked at 5-position and the galloyl is happen to be galactose. Finally, the peak **a6** was tentatively identified as cyanidin 3- *O*-(2″-*O*-galloyl-galactopyranoside)-5-*O*-rhamnoside (Cy3galloylGa5Rh). We only got the information about aglycone (*m/z* 287) for **a5** in MS, and judged it as cyanidin aglycone by UV absorption spectroscopy. Then **a5** was tentatively identified as cyanidin derivative. Owing to the low content in samples and little information of MS, **a10** could not identified exactly except for one anthocyanin.

#### Qualitative analysis for Flavonol and Chalcone

**Figure 4:**
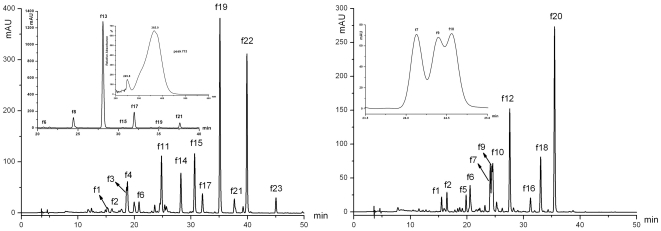
The HPLC profiles of glycosides of flavonol and chalcone in water lily petals (detected at 350 nm).

Using analysis by HPLC-DAD, 22 glycosides of flavonol (**f1**–**f12**, **f14**–**f23**) and one glycosides of chalcone (**f13**) (Fig. 4) have been detected by the characterization of UV-vis Absorption Spectroscopy for flavonol and chalcone. The data of HPLC-DAD and HPLC-ESI(+/−)-MS^2^ including retention time of HPLC, UV characteristic absorption wavelength, molecular ion, aglycone ion and some important fragment ions were summarized in Table S3.

Compared the references with characteristic of MS and UV Spectroscopy [30], 5 aglycones of flavonol and one chalcone have been found, containing 4 aglycones of flavonol: isorhamnetin (**f16** and **f20**), kaempferol (**f1**, **f7**, **f10**, **f15**, **f21** and **f22**), myricetin (**f1**, **f2**, **f3**, **f8**, **f9**, **f12** and **f14**) and quercetin (**f4**, **f5**, **f6**, **f11**, **f17**, **f18**, **f19** and **f23**); one aglycone of chalcone: chalcononaringenin (**f13**) (the chemical structure was illustrated in Fig. 3).

Through analysis by HPLC, 7 compounds were already known in comparison with standards and references: myricetin 3′-*O*-xyloside (My3′Xy) (**f9**); quercetin 3-*O*-rhamnoside (Qu3Rh) (**f11**); myricetin 3-*O*-(2″-acetylrhamnoside) (My3acetylRh) (**f14**); quercetin 3-*O*-(3″-acetylrhamnoside) (Qu3acetylRh) (**f17**); quercetin 3′-*O*-xyloside (Qu3′Xy) (**f18**); quercetin 3-*O*-(2″-acetylrhamnoside) (Qu3acetylRh) (**f19**) and kaempferol 3-*O*-(2″-acetylrhamnoside) (Km3-2″acetylRh) (**f22**). Except for **f1**, **f3** and **f4**, which were not identified exactly, the rest of the 12 compounds were detected in the petals of water lily for the first time in this study.

Under the negative ion modes, the relative abundance about aglycone ion ([Y_0_^−^]) and aglycone ion free radical ([Y_0_-H]^−.^) of flavonoid 3-*O*-glycoside and 7-*O*-glycoside was different. When glycosylation took place in 3-position, the relative abundance of [Y_0_-H]^−^. was higher than that of [Y_0_^−^], the situation is reverse when glycosylation happened to 7-position [31]. This conclusion further verified the structure of the known flavonoids (**f11**, **f14**, **f17** and **f19**), meanwhile we could suppose **f6** and **f23** to be quercetin 7-*O*-hexoside and quercetin 3-*O*-acetylhexoside. Only in one research, it had been reported that galactose glycosylated with flavonoid [21], so peak **f6** and **f23** were identified as quercetin 7-*O*-galactoside (Qu7Ga) and quercetin 3-*O*-acetylgalactoside (Qu3acetylGa) tentatively. As we all know, the characteristic of UV absorbance wavelength about the above two glycosides flavonoid was different, the band of flavonoid 7-*O*-glycoside caused bathochromic shifts compared with 3-*O*-glycoside [32]. As a result the structure of five compounds was identified as follows: **f8**: myricetin 3-*O*-galactoside (My3Ga), **f15**: kaempferol 3-*O*-galactoside (Km3Ga), **f16**: isorhamnetin 7-*O*-galactoside (Is7Ga), **f20**: isorhamnetin 7-*O*-xyloside (Is7Xy), **f21**: kaempferol 3-*O*-(3″-acetylrhamnoside) (Km3-3″acetylRh).

Although only one disaccharide has been isolated from leaves of the water lily *Nymphaea*×*marliacea* (white petals) [21], we could still not ensure the linking style between two monosaccharides, although the usual style was 1→2 and 1→6. Based on data of MS, the aglycone quercetin of **f5** was judged to be connected by two sugars: one hexose and one rhamnose, and quercetin was certainly glycosided with disaccharide because *m/z* 463 [M-H-146]^−^ was detected except for *m/z* 447 [M-H-162]^−^. The relative abundance of fragment ion *m/z* 303 [Y_0_^+^] was higher than that of *m/z* 463 [M+H-146]^+^, the connection type of glycosidic bond of disaccharide was 1→2 [33]. Compared the characteristic of UV absorbance of the known compounds (**f11**; **f17** and **f19**), **f5** was identified as quercetin 7-*O*-galactosyl-(1→2)-rhamnoside (Qu7GaRh) tentatively. As the same way, **f2** and **f7** were tentatively identified as myricetin 7-*O*-rhamnosyl-(1→2)-rhamnoside (My7RhRh) and kaempferol 7-*O*-galactosyl-(1→2)-rhamnoside (Km7GaRh) separately. Peak **f10** had fragment ions at *m/z* 449 [M+H-152]^+^ and *m/z* 315 [galloylhexose+H]^+^ (86), it illustrated that **f10** was acylated by gallic acid, and the galloyl connected with galactose. According to above information, the structure of **f10** was identified as kaempferol 7-*O*-galloylgalactosyl-(1→2)-rhanmoside (Km7galloylGaRh) provisionally.

With regard to acylated compounds, there were four components (**f14**, **f17**, **f19** and **f22**) isolated by Fossen et al., and the common acyl was acetyl and galloyl in water lily, so except for **f10**, **f21** and **f23**, the rest acylated peak **f12** was temporary identified as myricetin 3-*O*-galloylrhamnoside (My3galloylRh).

One glycoside of chalcone had been detected by the chromatogram monitored at 350 nm, depending on the characteristic of UV-vis spectrum, **f13** presented an intense absorption at 366 nm and a weak absorption at 250 nm [32]. The result of MS (*m/z*: 457[M+Na]^+^, 273[Y_0_^+^], 433[M-H]^−^, and 271[Y_0_^−^]) complyed with previous reports [34]. Therefore **f13** was identified as chalcononaringenin 2′-*O*-galactoside (Chal2′Ga), this compound was first reported in petals of water lily in this study and only existed in yellow flowers. Meanwhile it was also a principal component.

### Relationships between color parameters and flavonoid compositions

The flower color is associated with type, content and physicochemical property of pigment, the pH value of vacuole, the shape of epidermis cell and organization structure in petals. However the composition of pigments is the most important one. So choosing *L* ^*^, *a* ^*^, *b* ^*^, *C* ^*^ and *h* as dependent variable, 37 indexes included 34 contents of pigment components, TA (total anthocyanins content), TF (total flavonols and chalcones content) and CI (co-pigment index = TF/TA) as independent variable. The regression equations about the relationships between color parameters and flavonoids components were established to study the interactions between pigment composition and color formation by multiple linear regression (MLR) analysis [35]. Statistical results indicated as follows (n = 35, p<0.05):

*L* ^*^ = 61.074+0.032Qu3Rh+0.054Km7galloylGaRh - 0.031Dp3′galloylGa (R^2^ = 0.531)

*a* ^*^ = −3.113+0.040TA+0.065Qu3acetylGa (R^2^ = 0.850)

*b* ^*^ = −20.226−0.202Qu3acetylGa+0.007Chal2′Ga+0.016Qu3acetylRh (R^2^ = 0.673)

*C* ^*^ = 10.750+0.032TA+0.167Qu3acetylGa (R^2^ = 0.688)

*h* = −1.322+0.005Dp3galloylGa−0.001Dp3′galloylGa (R^2^ = 0.953)

From this MLR analysis, it can be seen that there are many factors affecting the color: TA; Dp3galloylGa; Dp3′galloylGa; Qu3Rh; Qu3acetylGa; Qu3acetylRh; Km7galloylGaRh and Chal2′Ga. TA and Qu3acetylGa had positive effects on the value of *a* ^*^ and *C* ^*^, whereas Qu3acetylGa and Qu3acetylRh had negative effects on the value of *b* ^*^, meanwhile Chal2′Ga had positive effects on the value of *b* ^*^. Km7galloylGaRh and Qu3Rh had positive effects on the value of *L* ^*^, but Dp3′galloylGa had negative effects on the value of *L* ^*^ and *h*. In addition Dp3galloylGa had a positive effect on the value of *h*. Based on those conclusions we can see increasing TA, Dp3′galloylGa and Qu3acetylGa, the value of *a* ^*^ and *C* ^*^ increased, but *L* ^*^ and *b* ^*^ decreased that means the flower colors change to be red and blue and much vivid. The compounds Chal2′Gal only exist in yellow water lily cultivars. And in the equation of *b* ^*^ the same result is gained: the higher contents of Chal2′Gal, the deeper of the yellow flower color. From the equations, we could see that increasing TA and the content of Qu3acetylGa the flower color would be more vivid.

### Comparison of anthocyanin components among cultivars

The main anthocyanins in the petals of tropic water lily were delphinidin glycosides, followed by cyanidin glycosides. Dp3′Ga was presented in all cultivars with blue colors, the highest amounts was detected in cultivar ‘Tai Guo Wang’ which the flower demonstrated blue purple (similar to Fig. 1E). Dp3Ga was only detected in ‘Huang Guan Zi', ‘34’ and ‘Fo Shou Lian’ which ranked the first three cultivars with the highest delphinidin derivates (Table S4, marked by boldface). It can be concluded that at 3-position of the B ring galactose was preferred to be linked, however, in tree peony [10], [11], *Lycoris longituba* [35] and lotus [36], glucose was abundant at the same position. It would be possibly unique for water lily plants, since flavonoids were thought to be evolutionarily adaptive for plants, the enzyme function for glycosylation of flavonoids might be also evolved in water lily. Within the blue group, Cy3Ga was the only one detected in the petal of ‘Huang Guan Zi’ with the highest TA value. The type of anthocyanins within flowers of ‘18’ (Fig. 1B), ‘27’ (Fig. 1C) and ‘Tai Guo Wang’ (Fig. 1I), the colors of which presented thin-blue, dark-blue and blue purple respectively, maybe due to the increased content of Dp3′Ga or TA value. It could be deduced that Dp3′Ga contributes most of the blue color formation in tropic water lily.

In contrast, in the amaranth group (represented by Fig. 1G), Dp3Ga was the only detected delphinidin derivatives with the highest amount. Compared with blue group, cultivars which were detected Dp3Ga presented amaranth, or detected Dp3′Ga for blue colors. In red group, the derivatives of Dp and Cy were more complicated, Dp3RhGa, Cy3Ga5Rh were also detected. ‘Albert Greenberg’ (Fig. 1D) and ‘Roxburgh’ (Fig. 1H) were illustrated with different colors, which can be deduced by the type of Dp and Cy derivatives and the amount of Cy derivative, the former contained Dp3RhGa, Dp3Ga, the latter with Dp3Ga, Dp3Rh, Dp3′Ga and Cy3Ga, although the content of Dp derivatives was close, the latter had the maximal amount of Cy derivatives.

To the best of our knowledge, besides glycosylation and hydroxylation, acylation also happened to tropic water lily. From Table S4 we can see 2″-galloyl-6″-acetyl presented in most cultivars of tropic water lily, but the highest amount existed in red group. It was obvious to know that 6″-acetyl and 2″-galloyl-6″-oxalyl were only detected in amaranth and red group respectively, but the contents were not high. 2″-galloyl was detected in amaranth, red group and some cultivars of blue group. No anthocyanins were detected within white and yellow group. These two groups might be useful breeding materials when crossing with the other groups to produce novel colors of cultivars.

### Putative flavonoid biosynthesis pathway of tropical water lily

**Figure 5:**
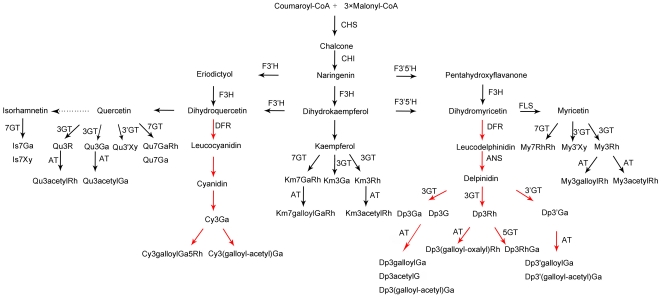
Putative flavonoid biosynthesis pathway related to flower color of tropical water lily. The red arrows indicate the biosynthesis pathway of anthocyanins, the dotted arrows indicate uncertain reaction. Cy: cyanidin; Dp: delphinidin; Qu: quercetin; Km: kaempferol; My: myricetin; Is: isorhamnetin; Ga: glactoside; G: glucoside; Rh: rhamnoside; CHS: chalcone synthase; CHI : chalcone isomerase, F3H: flavonoid 3-hydroxylase; F3′H: flavonoid 3′-hydroxylase; F3′5′H: flavonoid 3′,5′-hydroxylase; DFR: dihydroflavonol reductase; FLS: flavonol synthase; ANS: anthocyanidin synthase; GT: flavonoid glycosyltransferase; AT: acyltransferase; MT: methyltransferase; CoA: acetyl coenzyme A.

Flavonoid especially anthocyanin biosynthesis pathway is one of the most extensively studied pathway of plant secondary products [37]. With the detected component of flavonoids in tropical water lily, we deduced the putative flavonoid biosynthesis pathway relevant to flower color (Fig. 5). It rooted from coumaroyl-CoA and malonyl-CoA, with the enzymes of CHS, CHI and F3H, finally, synthesized dihydrokaempferol. Then it was divided into five sub-pathway for synthesis of anthocyanins and flavonols. With the function of F3′H, F3′5′H, DFR and ANS or FLS, anthocyanidins (cyanidin and delpinidin) and aglycone of flavonols were obtained, which finished the first important modification of hydroxylation of flavonoids. The obtained secondary metabolites were glycosylated by glycosyltransferase at different position in tropical water lily. Flavonoids including anthocyanins exist in glycosylated form in vivo, although most anthocyanins are glycosylated at 3-*O*-position and often at 5-*O*-position, the former is a perquisite for further modifications including second glycosylation, acylation and methylation [37]. In tropical water lily, glycosylation at 3-*O*-position was most often, and the galactose was preferred to be added to the anthocyanidins. The selection of different substrates or different position for glycosylation in different plants may shed light on the related function of glycosyltransferase during the evolution of plants or the enzymes themselves. It is still need to be further studied for the function of this kind of enzymes. Modification of flavonoids by hydroxylation, glycosylation, methylation and acylation played an important role for flower color formation. In tropical water lily, the glycosylated flavonoids were further modified by acyltransferase. It is presumed that acylation with aromatic organic acid to contribute for stabilization of flavonoids due to intra- and/or inter-molecular stacking as co-pigmentation. Most acylated flavonoids in the petals of tropical water lily may intensify blue color as a bathochromic effect. The exact flavonoid biosynthesis pathway relevant to flower color of tropical water lily was still need to be confirmed by more molecular biology or biochemical evidences.

## Discussion

As a perennial plant, tropical water lily is characteristic of some unique biological features such as the special flower colors like blue, violet and bluish purple and some cultivars blooming at night. In general, tropical water lily spreads all over the world and its flower colors are diverse. In this study, using an HPLC-DAD/MS analytical method to characterize flavonoids of water lily petals within 50 min, finally 34 flavonoids have been identified at 525 nm and 350 nm, respectively, in which three anthocyanins, twelve flavonols and one chalcone were discovered for the first time in petals of water lily.

Compared the graphs of HPLC with the results of MLR analysis, we could see that Dp3′galloyl-acetylGa (**a7**) and Dp3galloyl-acetylGa (**a9**) were the main anthocyanins in those 35 cultivars, but they did not take part in the MLR analysis and in reverse Dp3′galloylGa (**a2**) and Dp3galloylGa (**a3**) replaced them. Meanwhile compared with **a2** compound, **a7** owned an acetyl group at 6″ position, so did **a9**. However, there were no similar regularities in flavonols, so the results of MLR analysis were synthetic with anthocyanins, flavonols and chalcones. A correlation analysis indicated that the *L* ^*^ value increased with increasing proportions of Km7galloylGaRh (**f10**) and Qu3Rh (**f11**) and the reduction of the contents of **a2**. At the same time we could see that increasing the contents of TA, Qu3acetylGa (**f23**) and **a2**, the value of *a* ^*^ and *C* ^*^ increased while *L* ^*^ and *b* ^*^ decreased, which means that the flower colors changed to be red, blue and much vivid. The compound Chal2′Ga (**f13**) only existed in yellow water lily cultivars, and in the equation of *b* ^*^, the same result was gained that the higher contents of **f13**, the deeper of the yellow flower color. So it was the indispensable compound in formation of the yellow flower in water lily.

The relationships between color parameters and flavonoid compositions showed that many kinds of compounds played an important role on color formation. To characterize the mechanism on flower color formation of special blue and bluish violet which hardy water lily lacks was necessary because it could provide powerful evidences for the ornamental breeding of hardy water lily with blue colors and help to classify cultivars of *Nymphaea* through phytochemical analysis. At the same time, a HPLC fingerprinting database of water lily cultivars could be established with the flavonoids composition data for discriminating cultivars.

Anthocyanins not only contribute to flower colors, but also play a vital role on bioactivity including anti-oxidant, anti-cancer, anti-allergic and anti-ulcer, therefore they were used as diets in cancer therapy and prevention [38]. Among five anthocyanidins, delphinidin and cyanidin inhibited LPS-induced COX-2 expression, but pelargonidin, peonidin and malvidin did not, the structure-activity relationship suggested that the ortho-dihydroxyphenyl structure of anthocyanidins on the B-ring appears to be related to the inhibitory actions, especially delphinidin as the most potent inhibitor [39]. Delphinidin also protects human HaCaT keratinocytes and mouse skin against UVB-mediated oxidative stress and apoptosis [40]. In tropical water lily, cyanidin and delphinidin are the main anthocyanidins, which may be judged for the high bioactivity for developing functional food or medicine materials.

Other flavonoids were also important components for antioxidant activity, among which quercetin and kaempferol have high antioxidant activity; apigenin and chalcononaringenin or its derivates demonstrated high antioxidant ability due to hydroxylation of B-ring at 4-position [11]. In yellow group of tropical water lily, the content of Chal2′Ga accounted for 80% of the total amount of other flavonoids (TF). However, in blue group the amount of qucercetin was about 87.7% of TF, in amaranth group the amount of kaempferol reached 92.8% of TF. These abundant flavonoids will be good resource for bioactivity for future utilization.

Genes for enzymes involved in the synthesis of flavonoid for flower colors were cloned in model plants such as petunia, arabidopsis, rice etc. People tried to change or create novel flower colors through over-expressed or knocked down some genes related to this pathway [41]. The deduced pathway in this study will help to understand its synthesis of flavonoids and to clone the related genes for molecular breeding of tropical water lily with novel flower colors or studies on molecular mechanism for the formation of flower colors.

## Materials and Methods

### Standards and solvents

Malvidin 3,5-di-*O*-glucoside chloride (Mv3G5G) was purchased from Extrasynthese (Genay, France). quercetin 3-*O*-rutinoside (rutin) was obtained from the National Institute for the Control of Pharmaceutical and Biological Products (Beijing, China). Acetonitrile and methanol used for high-performance liquid chromatography-photodiode array detection (HPLC-DAD) and electrospray ionization multistage mass spectrometry (ESI-MS^n^) analysis were of chromatographic grade and obtained from Alltech Scientific (Beijing, China). Trifluoroacetic acid (TFA; ≥99%) was purchased from Merck (Darmstadt, Germany). Methanol, formic acid and hydrochloric acid of analytical grade were obtained from Beijing Chemical Works (Beijing, China). HPLC-grade water was purchased from a Milli-Q System (Millipore, Billerica, MA, USA).

### Plant Material and Petal Color Measurement

Petals of 35 tropical water lily cultivars were all collected in 2010 at Beijing Botanical Garden, Institute of Botany, the Chinese Academy of Sciences (Long. 39°48′N, Lat. 116°28′E, Alt. 76 m), Beijing, China. These cultivars introduced from all around the world have been planted in the same-sized containers (diameter, 40 cm; height, 30 cm) with two cultivars in each small pond (length×width×depth: 140 cm×140 cm×70 cm) in Beijing Botanical Garden for more than 3 years under the same cultivated conditions like fertilization, irrigation, disease and insect prevention and so on. Petals were harvested at the first blooming day.

**Figure 6:**
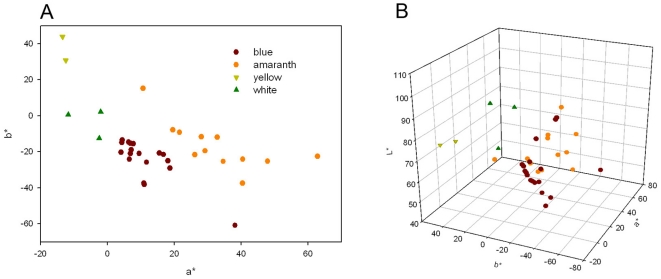
Flower color distribution of Tropical water lily cultivars in coordinate systems of bivariate (*a* ^*^ and *b* ^*^) (A) and trivariate (*a* ^*^, *b* ^*^, and *L* ^*^) (B), respectively. The flower colors were identified by the RHSCC value.

The fresh petals were compared to Royal Horticultural Society Colour Chart (RHSCC) and the color parameters were measured with a spectrophotometer NF333 (Nippon Denshoku Industries Co. Ltd., Japan). Then petals from 35 cultivars were stored in refrigerator at −40°C for future analysis. In this study, 35 cultivars were classified into four groups roughly in terms of floral colors (Table 2). CIE 1976 *L* ^*^ *a* ^*^ *b* ^*^ (CIELAB), a software package, is used to measure different aspects of a flower color and takes into account all aspects of the color described by *L* ^*^, *a* ^*^, and *b* ^*^ parameters [42]. The *L* ^*^ describes the lightness of the color, ranging from black (*L* ^*^ = 0) to white (*L* ^*^ = 100). The *a* ^*^ negative and positive are for green and red, and the *b* ^*^ negative and positive are for blue and yellow, respectively [35]. The color coordinates measured were shown in Fig. 6: the *L* ^*^ values ranged from 44.36 to 99.92, *a* ^*^ values from −13.40 to 62.91 and *b* ^*^ values from −60.86 to 15.24. Two new parameters, Chroma [*C* ^*^ = (*a* ^*2^+*b* ^*2^)^1/2^] and hue angle [*h* = arctan b^*^/a^*^ ], were derived from *a* ^*^ and *b* ^*^. The Chroma parameter describes the saturation of the color. The *C* ^*^ value is higher, the color is more saturated. The hue angle parameter describes the hue of the color. Hue angle values are stepped counterclockwise across a continuously fading hue circle, some special colors' values of which are remarkable: magenta (0°/360°), yellow (90°), bluish-green (180°) and blue (270°) [42], [43].

**Table 2:** Petal colors and color parameters of 35 cultivars of water lily.

Sample^1^	Colour	RHSCC^2^	CIE *L* ^*^ *a* ^*^ *b* ^*^ 3					TA^4^	TF^4^	CI^5^
			*L* ^*^	*a* ^*^	*b* ^*^	*C* ^*^	*h*			
Nan Fei waterlily	blue	97C	64.89	6.38	−14.63	15.96	−1.16	0.19	3.23	16.80
Zhong Hua Lan	blue	97A	60.57	7.17	−18.95	20.27	−1.21	0.43	3.82	8.85
Hu Die Lan	blue	97A	60.40	6.92	−20.90	22.03	−1.25	0.39	3.50	9.08
13	blue	92C	90.19	10.92	−37.32	38.89	−1.29	0.21	5.01	23.50
Colorata	blue	92C	90.99	11.09	−38.11	39.70	−1.29	0.20	5.36	26.17
Zanaibar	blue	92C	62.47	7.78	−15.60	17.48	−1.10	0.21	3.95	19.00
18	blue	91C	64.01	6.92	−15.30	17.07	−1.11	0.24	2.90	12.04
Bao Luo Lan	blue	97A	53.56	11.76	−25.83	28.39	−1.14	0.54	4.70	8.73
22	blue	97B	58.84	9.54	−20.93	23.00	−1.14	0.30	3.43	11.48
23	blue	97C	67.92	4.39	−14.81	15.45	−1.29	0.22	5.69	26.31
27	blue	92A	48.96	18.72	−29.11	34.62	−1.00	0.42	3.98	9.41
29	blue	97C	81.17	6.63	−24.07	24.99	−1.28	0.16	3.31	21.22
34	mazarine	91A	57.07	15.57	−20.67	25.92	−0.93	0.60	3.65	6.04
Fo Shou Lian	bluish violet	90C	44.36	18.18	−25.02	30.95	−0.94	0.60	3.85	6.46
Huang Guan Zi	bluish violet	N88D	63.18	16.85	−21.53	27.35	−0.91	0.81	3.95	4.87
42	blue	97A	68.58	4.45	−13.51	16.44	−1.23	0.18	4.24	23.99
Lan He	blue	97C	70.73	4.10	−20.24	20.78	−0.49	0.09	5.29	59.81
Tai Guo Wang	blue	92A	59.95	38.20	−60.86	71.90	−1.01	0.56	2.77	4.99
Qi Feng	amaranth	75B	77.01	26.19	−21.61	34.64	−0.61	0.52	3.05	5.85
9	amaranth	N66D	56.90	28.11	−11.71	30.48	−0.40	0.86	5.23	6.11
10	amaranth	N66D	64.84	34.67	−25.34	46.91	−0.54	0.85	4.37	5.12
Ruby	amaranth	N66D	74.15	29.27	−19.43	33.74	−0.51	0.88	4.08	4.61
Pink pearl	amaranth	75B	65.86	19.60	−7.88	21.13	−0.38	0.56	3.84	6.89
37	amaranth	73A	56.05	32.90	−11.90	35.00	−0.35	0.74	4.12	5.59
47	amaranth	72C	61.88	47.98	−25.19	54.90	−0.45	1.25	4.75	3.81
48	amaranth	N74D	74.67	40.51	−37.48	55.19	−0.75	0.80	5.57	6.95
49	amaranth	75B	63.037	21.615	−9.149	23.483	−0.4023	0.404	4.112	10.183
Redflagsong	red	65A/N57D	86.54	40.52	−24.18	47.25	−0.55	1.23	4.78	3.88
Albert Greenberg	ocher red	165D	65.86	10.68	15.24	19.02	0.96	0.36	6.55	18.03
Roxburgh	dark red	67A	47.57	62.91	−22.53	66.84	−0.34	1.42	3.19	2.25
Ai Ji Bai	white	157C	116.92	−11.70	0.62	12.54	0.02	0.00	3.00	∞
20	light blue	92D	76.54	−2.00	2.12	3.05	−0.96	0.00	6.40	∞
He Hua water lily	light blue	NN155B	96.94	−2.45	−12.47	12.71	1.38	0.00	6.40	∞
Eldorado	yellow	1C	80.14	−12.44	30.84	33.25	−1.19	0.00	7.93	∞
Mo Xi Ge Huang	yellow	3C	77.37	−13.40	43.99	45.99	−1.27	0.00	7.45	∞

1 The name of water lily cultivar;

2 RHSCC: Royal Horticultural Society Colour Chart;

3 *L* ^*^, lightness; *a* ^*^, *b* ^*^, chromatic components; *C* ^*^, chroma, *C* ^*^ = (*a* ^*2^+*b* ^*2^)^1/2^; *h*, hue angle (u), *h* = arctan (*b* ^*^/*a* ^*^);

4 TA: total anthocyanins; TF: total flavonols and chalcones in mg per 1 g fresh petals;

5 CI: copigment index = TF/TA; “∞”: means samples without glycosides of anthocyanin.

### Preparation of standard solutions

The standards of Mv3G5G and rutin weighted accurately were dissolved in 0.1% (V/V) HCl-methanol and methanol, respectively, and then diluted to a series of concentrations (mg/mL): 0.01, 0.025, 0.05, 0.1, 0.2, 0.4, 0.6 and 0.8.

### Extraction and Preparation of the Flavonoids

The extraction method of flavonoids was modified from that of Yang et al. [36]. Approximately 0.6 g of frozen petal was powdered in liquid nitrogen with mortars and pestles and extracted for the first time with 3 mL 70% (V/V) methanol aqueous solution containing 0.1% HCl shaken in a QL-861 vortex (Kylinbell Lab Instruments, Jiangsu, China), sonicated in KQ-500DE ultrasonic cleaner (Ultrasonic instruments, Jiangsu Kunshan, China) at 20°C for 20 min, centrifuged in SIGMA 3K30 (SIGMA centrifugers, Germany) (12000 rpm, 10 min), and the supernatant was collected. Additional 2 mL and 1 mL extraction solution was supplemented to the residue, and repeated aforesaid operation for second and third times. All extract was pooled and filtrated through 0.22 µm reinforced nylon membrane filters (Shanghai ANPEL, Shanghai, China) before the HPLC-DAD and HPLC-MS analyses. Three replicates were performed for each sample.

### HPLC-DAD Systems and Conditions

HPLC analysis was performed on a Dionex (Sunnyvale, CA, USA) system including a P680 HPLC pump, an UltiMate 3000 autosampler, a TCC-100 thermostated column compartment and a PDA100 photodiode array detector. The liquid chromatograph was equipped with an ODS-80Ts QA C18 column (250 mm×4.6 mm i.d., Tosoh, Tokyo, Japan), which was protected with a C18 guard cartridge (Shanghai ANPEL Scientific Instrument, Shanghai, China). Eluent A was 10% formic acid aqueous solution; eluent B was 0.1% formic acid in acetonitrile [44]. A gradient elution as follows was used: 8% B at 0 min, 18% B at 15 min, 23% B at 25 min, 40% B at 45 min, 8% B at 50 min. The flow rate was 0.8 mL·min^−1^ and aliquots of 10 µL of analytes were injected. Column temperature was maintained at 35°C for all analyses. Chromatograms were acquired at 520 and 350 nm for anthocyanins and other flavonoids, respectively, and DAD data were recorded from 200 to 800 nm.

### HPLC-MS System and Conditions

HPLC-ESI-MS^n^ analysis for anthocyanins and other flavonoids were carried out in an Agilent-1100 HPLC system equipped with a UV detector and a LC-MSD Trap VL ion-trap mass spectrometer via an ESI source (Agilent Technologies, Palo Alto, CA, USA). The HPLC separation conditions were the same as mentioned above. The MS conditions were as follows: anthocyanins were adopted in positive-ion (PI) mode and other flavonoids were employed in negative-ion (NI) mode. ESI was performed by using the following conditions: capillary voltage, 4.0 kV; a nebulization pressure, 241.3 kPa; and a gas (N_2_) temperature, 350°C; flow rate, 8.0 L·min^−1^. Capillary offset and exit voltage were 77.2 V and 127.3 V, respectively for PI, and −77.2 V and −127.3 V separately for NI. MS spectrum was recorded over the range from *m/z* 100 to 1000.

### Statistical Analysis

Correlations between petal color parameters and flavonoid compositions of individual varieties were analyzed by a multiple linear regression (MLR) (SPSS17.0 Inc., CHI, IL, USA) with stepwise method.

## Supporting Information

Figure S1 The graphs of glycosides of flavonoids (350 nm) separated between longer column (250 mm) (a) and shorter column (150 mm) (b).
(TIF)

Table S1 Linearity of response for Mv3G5G and rutin using the optimized method. Calibration fitting: y = kx+m^1^. ^1^ In the regression equation y = kx+m, y refers to the peak area, x is concentration of the standard substances (µg/mL), r^2^ is the correlation coefficient of the equation.
(DOC)

Table S2 Intra- and inter-day precision of 31 main flavonoids in the extract of water lily petals by HPLC-DAD.
(DOC)

Table S3 HPLC-DAD and HPLC-ESI-MS^n^ analysis as well as the structure characterization and tentative identification of glycosides of flavonol and chalcone in petals of water lily.
(DOC)

Table S4 The mean content (µg/g) of petal anthocyanin in 35 tropic water lily varieties.
(DOC)
