# Supplementary figures and images for: Relationship between the Composition of Flavonoids and Flower Colors Variation in Tropical Water Lily (Nymphaea) Cultivars
Source: PLoS One. 2012 Apr 2;7(4):e34335. doi: 10.1371/journal.pone.0034335 (PMC3317528; doi:10.1371/journal.pone.0034335)

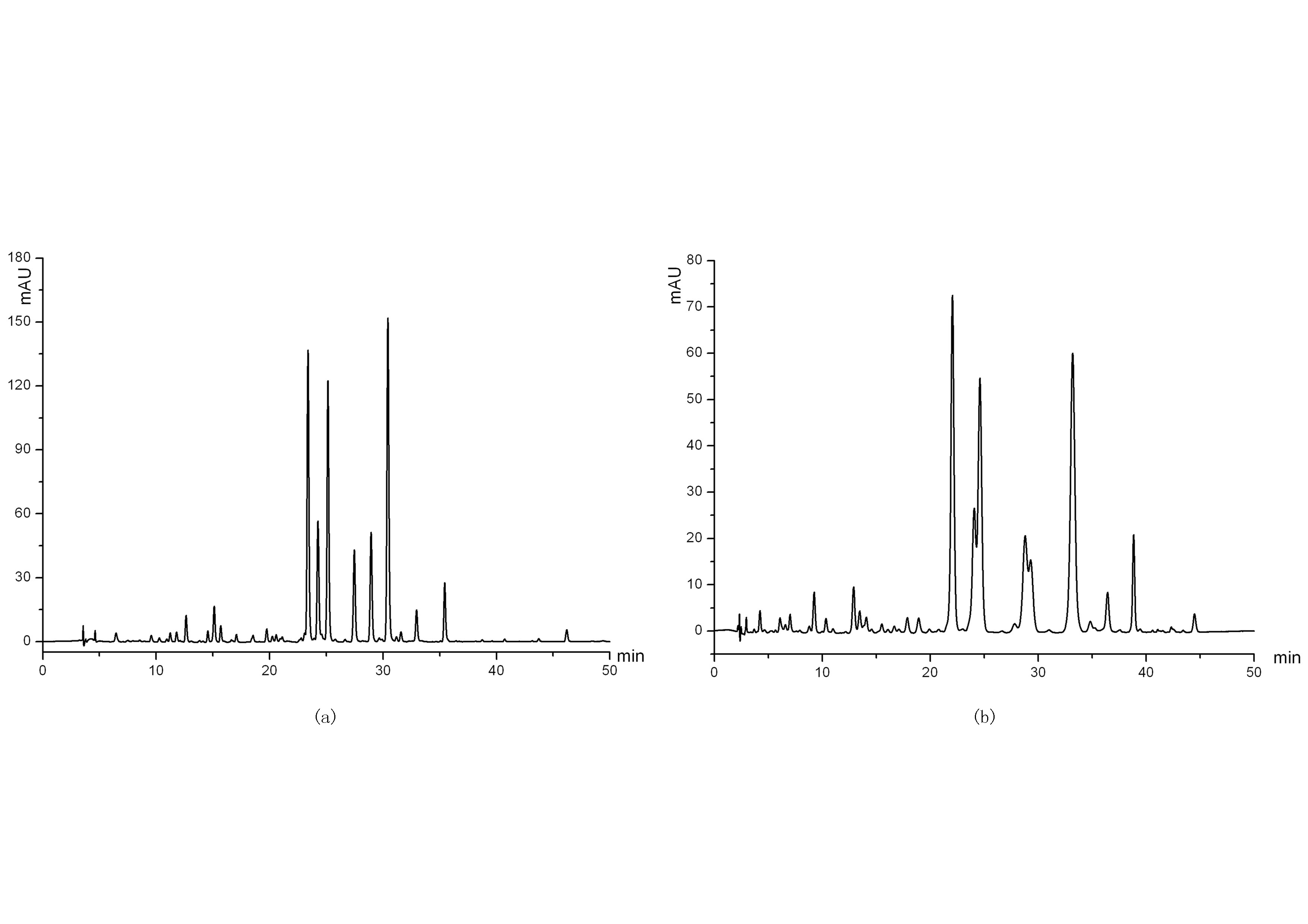

Supplement: Figure S1 — The graphs of glycosides of flavonoids (350 nm) separated between longer column (250 mm) (a) and shorter column (150 mm) (b). (TIF) [file pone.0034335.s001.tif]
