# Supplementary material for: Relationship between the Composition of Flavonoids and Flower Colors Variation in Tropical Water Lily (Nymphaea) Cultivars
Source: PLoS One. 2012 Apr 2;7(4):e34335. doi: 10.1371/journal.pone.0034335 (PMC3317528; doi:10.1371/journal.pone.0034335)
Supplement: Table S1 — Linearity of response for Mv3G5G and rutin using the optimized method. Calibration fitting: y = kx+m1. 1 In the regression equation y = kx+m, y refers to the peak area, x is concentration of the standard substances (µg/mL), r2 is the correlation coefficient of the equation. (DOC) [file pone.0034335.s002.doc]

**Table S1.** Linearity of response for Mv3G5G and rutin using the optimized method. Calibration fitting: y=kx+m^1^.

| **standards** | **Slope(k)**  **(mean±SD^2^, n = 3)** | **Intercept (m)**  **(mean±SD^2^, n=3)** | **Regression r^2^(n=5)** | **LOD^3^(μg/mL)** | **LOQ^3^(****μg/mL)** |
| --- | --- | --- | --- | --- | --- |
| rutin | 0.4495±0.0001 | -1.3753±0.0680 | 0.9986 | 0.4537 | 1.5124 |
| Mv3G5G | 0.5132±0.0002 | -4.3030±0.1230 | 0.9998 | 0.7193 | 2.3977 |

^1^ In the regression equation y=kx+m, y refers to the peak area, x is concentration of the standard substances (μg/mL), r^2^ is the correlation coefficient of the equation;

^2^ Standard deviation is abbreviated as SD;

^3^ LOD (the limit of detection, S/N = 3) and LOQ (limit of quantification, S/N = 10) were expressed in concentration unit with injection volume 10μL.
