# Supplementary material for: Relationship between the Composition of Flavonoids and Flower Colors Variation in Tropical Water Lily (Nymphaea) Cultivars
Source: PLoS One. 2012 Apr 2;7(4):e34335. doi: 10.1371/journal.pone.0034335 (PMC3317528; doi:10.1371/journal.pone.0034335)
Supplement: Table S2 — Intra- and inter-day precision of 31 main flavonoids in the extract of water lily petals by HPLC-DAD. (DOC) [file pone.0034335.s003.doc]

**Table S2.** Intra- and inter-day precision of 31 main flavonoids in the extract of water lily petals by HPLC-DAD.

| **Compound^1^ No.** | **Intra-day precision (n=3)** | | **Inter-day precision (n=6)** | |
| --- | --- | --- | --- | --- |
|  | **Content (μg/g)^2^** | **R.S.D. (%)^3^** | **Content (μg/g)^2^** | **R.S.D. (%)^3^** |
| a1 | 77.05 | 0.65 | 79.50 | 0.20 |
| a2 | 200.05 | 0.28 | 195.00 | 1.14 |
| a3 | 115.21 | 0.67 | 114.76 | 2.12 |
| a4 | 193.20 | 0.35 | 201.91 | 0.73 |
| a5 | 87.81 | 0.26 | 91.01 | 1.49 |
| a6 | 114.19 | 0.07 | 121.73 | 2.22 |
| a7 | 323.51 | 0.03 | 347.39 | 2.23 |
| a9 | 208.72 | 0.44 | 199.00 | 3.34 |
| a10 | 86.33 | 0.32 | 92.65 | 3.14 |
| a11 | 148.09 | 0.11 | 152.06 | 3.21 |
| f1 | 102.40 | 0.24 | 87.96 | 2.45 |
| f2 | 130.15 | 0.48 | 110.11 | 2.11 |
| f3 | 83.94 | 0.54 | 80.89 | 1.01 |
| f5 | 117.42 | 0.58 | 115.36 | 3.46 |
| f6 | 185.83 | 0.45 | 170.80 | 1.38 |
| f7 | 418.74 | 0.32 | 358.74 | 0.03 |
| f8 | 840.70 | 0.14 | 821.65 | 1.70 |
| f10 | 408.95 | 0.08 | 381.75 | 2.57 |
| f11 | 276.57 | 0.05 | 280.84 | 0.98 |
| f12 | 724.25 | 0.41 | 693.33 | 2.17 |
| f13 | 3466.03 | 0.55 | 3493.35 | 1.87 |
| f14 | 99.32 | 0.32 | 99.30 | 0.99 |
| f15 | 89.82 | 0.33 | 89.89 | 0.43 |
| f16 | 97.89 | 0.54 | 91.26 | 3.35 |
| f17 | 1600.63 | 0.39 | 1483.85 | 3.06 |
| f18 | 348.90 | 0.55 | 328.24 | 0.73 |
| f19 | 117.47 | 0.36 | 119.23 | 2.20 |
| f20 | 1201.54 | 0.30 | 1207.30 | 2.79 |
| f21 | 498.93 | 0.25 | 492.40 | 1.45 |
| f22 | 50.32 | 0.30 | 50.24 | 1.16 |
| f23 | 34.90 | 0.74 | 36.59 | 2.88 |

^1^ Compounds a1-a11 were quantified by Mv3G5G, while compounds f1-f23 by rutin;

^2^ The parameter discussed is a recalculated content of the studied compounds, described in μg per 1 g fresh petals;

^3^ RSD: relative standard deviation.
