# Supplementary material for: Relationship between the Composition of Flavonoids and Flower Colors Variation in Tropical Water Lily (Nymphaea) Cultivars
Source: PLoS One. 2012 Apr 2;7(4):e34335. doi: 10.1371/journal.pone.0034335 (PMC3317528; doi:10.1371/journal.pone.0034335)
Supplement: Table S3 — HPLC-DAD and HPLC-ESI-MSn analysis as well as the structure characterization and tentative identification of glycosides of flavonol and chalcone in petals of water lily. (DOC) [file pone.0034335.s004.doc]

**Table S3.** HPLC-DAD and HPLC-ESI-MS^n^ analysis as well as the structure characterization and tentative identification of glycosides of flavonol and chalcone in petals of water lily.

| **No**^1^**.** | **Identifacation/tentative  identification** | **tR（min）** | **λ_max_（nm）** | **EST-PI MS/MS^2^（m/z）** | **EST-NI MS/MS^2^（m/z）** | **References** |
| --- | --- | --- | --- | --- | --- | --- |
| f1 | kaempferol and myricetin derivatives | 15.52 | 263,358 | 287[Y_0_^+^](81),319[Y_0_^+^](38) | 285[Y_0_^-^](34),317[Y_0_^-^](6) |  |
| **f2** | myricetin 7-*O*-rhamnosyl-(1→2)-rhamnoside | 16.46 | 267,360 | 633[M+Na]^+^(23),465[M+H]^+^(10),319[Y_0_^+^](40) | 317[Y_0_^-^](6) |  |
| f3 | myricetin derivatives | 18.62 | 263,350 | 319[Y_0_^+^] | 317[Y_0_^-^] |  |
| f4 | quercetin derivatives | 18.77 | 254,365 | 303[Y_0_^+^](100) | 301[Y_0_^-^] |  |
| **f5** | quercetin 7-*O*-galactosyl-(1→2)-rhamnoside | 19.86 | 257,356 | 633[M+Na]^+^(36),465[M+H-146]^+^(22),303[Y_0_^+^](100) | 609[M-H]^-^(100),301[Y_0_^-^](5) |  |
| **f6** | quercetin 7-*O*-galactoside | 20.56 | 272,354 | 487[M+Na]^+^(15),303[Y_0_^+^](56) | 463[M-H]^-^(26),301[Y_0_^-^](4), 300[Y_0_-H]^-.^(1) |  |
| **f7** | kaempferol 7-*O*-galactosyl-(1→2)-rhamnoside | 24.13 | 266,348 | 617[M+Na]^+^(37),449[M+H-146]^+^(16),287[Y_0_^+^](100) | 593[M-H]^-^(100),285[Y_0_^-^](21) |  |
| **f8** | myricetin 3-*O*-galactoside | 24.36 | 263,349 | 481[M+H]^+^(2),319[Y_0_^+^](100) | 479[M-H]^-^(25),317[Y_0_^-^](5) |  |
| f9 | myricetin 3'-*O*-xyloside | 24.40 | 254,305,366 | 473[M+Na]^+^(5),451[M+H]^+^(94),319[Y_0_^+^](100) | 449[M-H]^-^(64),317[Y_0_^-^](5) | Fossen Torgils et al.,1998 |
| **f10** | kaempferol 7-*O*-galloylgalactosyl-(1→2)-rhanmoside | 24.56 | 268,350 | 769[M+Na]^+^(28),601[M+H-146]^+^(14),449[M+H-152]^+^(6), 315[galloylhexose+H]^+^(86),287[Y_0_^+^](65) | 745[M-H]^-^(100),285[Y_0_-]^-^(14) |  |
| f11 | quercetin 3-*O*-rhamnoside | 24.79 | 257,348 | 471[M+Na]^+^(15),303[Y_0_^+^](100) | 447[M-H]^-^(100),301[Y_0_^-^](7) | Fossen Torgils et al.,1999 |
| **f12** | myricetin 3-*O*-galloylrhamnoside | 27.57 | 252,356 | 617[M+H]^+^(5),465[M+H-152]^+^(100),319[Y_0_^+^](3) | 463[M-H]^-^(100),317[Y_0_^-^](4) | Fossen Torgils et al.,1999 |
| f13 | chalcononaringenin 2'-*O*-galactoside | 28.09 | 250,366 | 457[M+Na]^+^(100),273[Y_0_^+^](74) | 433[M-H]^-^(100),271[Y_0_^-^](10) |  |
| f14 | myricetin 3-(2"-acetylrhamnoside) | 28.22 | 263,350 | 529[M+Na]^+^(99),319[Y_0_^+^](46) | 505[M-H]^-^(100),317[Y_0_^-^](5),316[Y_0_-H]^-.^(6) |  |
| **f15** | kaempferol 3-*O*-galactoside | 30.65 | 265,343 | 471[M+Na]^+^(10),287[Y_0_^+^](100) | 447[M-H]^-^(2),285[Y_0_^-^](23) |  |
| **f16** | isorhamnetin 7-*O*-galactoside | 31.24 | 268,350 | 501[M+Na]^+^(9),479[M+H]^+^(100),317[Y_0_^+^](25),163[B_1_^+^](6) | 477[M-H]^-^(93),315[Y_0_^-^](12) |  |
| f17 | quercetin 3-(3"-acetylrhamnoside) | 32.04 | 257,348 | 513[M+Na]^+^(100),303[Y_0_^+^](49), 189[acetylrhamnoside+H]^+^(8) | 489[M-H]^-^(100),447[M-H-42]^-^(1), 301[Y_0_^-^](4), 300[Y_0_-H]^-.^(5) | Fossen Torgils et al.,1999 |
| f18 | quercetin 3'-*O*-xyloside | 33.03 | 254,366 | 457[M+Na]^+^(4),435[M+H]^+^(100),303[Y_0_^+^](78) | 433[M-H]^-^(97),301[Y_0_^-^](29) | Fossen Torgils et al.,1998 |
| f19 | quercetin 3-(2"-acetylrhamnoside) | 35.13 | 257,348 | 513[M+Na]^+^(31),449[M+H-146]^+^(8),303[Y_0_^+^](10) | 489[M-H]^-^(100),301[Y_0_^-^](3),300[Y_0_-H]^-.^(5) | Fossen Torgils et al.,1999 |
| **f20** | isorhamnetin 7-*O*-xyloside | 35.50 | 252,268,352 | 471[M+Na]^+^(7),449[M+H]^+^(100),317[Y_0_^+^](49) | 447[M-H]^-^(100),315[Y_0_^-^](21) |  |
| **f21** | kaempferol 3-(2"-acetylrhamnoside) | 37.68 | 265,342 | 497[M+Na]^+^(100),475[M+H]^+^(3),433[M+H-42]^+^(3), 287[Y_0_^+^](54),189[acetylrhamnoside+H]^+^(9) | 473[M-H]^-^(21),285[Y_0_^-^](32) | Fossen Torgils et al.,1999 |
| f22 | kaempferol 3-*O*-(2"-acetylrhamnoside) | 39.89 | 265,343 | 497[M+Na]^+^(100),287[Y_0_^+^](6) | 473[M-H]^-^(37),285[Y_0_^-^](37),284[Y_0_-H]^-.^(11) |  |
| **f23** | quercetin 3-*O*-acetylgalactoside | 45.03 | 257,348 | 529[M+Na]^+^(8),303[Y_0_^+^](10),163[B_1_^+^](13) | 505[M-H]^-^(1),301[Y_0_^-^](3),300[Y_0_-H]^-.^(3) |  |

^1^: The bold numbers of compounds were reported for the first time in tropic water lily
