# Supplementary material for: Relationship between the Composition of Flavonoids and Flower Colors Variation in Tropical Water Lily (Nymphaea) Cultivars
Source: PLoS One. 2012 Apr 2;7(4):e34335. doi: 10.1371/journal.pone.0034335 (PMC3317528; doi:10.1371/journal.pone.0034335)
Supplement: Table S4 — The mean content (µg/g) of petal anthocyanin in 35 tropic water lily varieties. (DOC) [file pone.0034335.s005.doc]

**Table S4.** The mean content (μg/g) of petal anthocyanin in 35 tropic water lily varieties.

| **Sample^1^** | **Dp^2^** | | | | | **Cy^2^** | | | **Aglycone^2^** | | | **Glycoside^2^** | | | | | | **Acyl^2^** | | |
| --- | --- | --- | --- | --- | --- | --- | --- | --- | --- | --- | --- | --- | --- | --- | --- | --- | --- | --- | --- | --- |
|  | **3RhGa** | **3Ga** | **3G** | **3Rh** | **3'Ga** | **3Ga5Rh** | **3Ga** | **De** | **Dp** | **Cy** | **3Ga** | **3G** | **3'Ga** | **3Rh** | **3RhGa** | **3Ga5Rh** | **2"galloyl** | **6"acetyl** | **2"galloyl 6"acetyl** | **2"galloyl 6"oxalyl** |
| **Blue** |  |  |  |  |  |  |  |  |  |  |  |  |  |  |  |  |  |  |  |  |
| Nan Fei waterlily | - | - | - | - | 192.27 | - | - |  | 192.27 | - | - | - | 192.27 | - | - | - | - | - | 192.27 | - |
| Zhong Hua Lan | - | - | - | - | 431.66 | - | - |  | 431.66 | - | - | - | 431.66 | - | - | - | 123.82 | - | 307.84 | - |
| Hu Die Lan | - | - | - | - | 385.48 | - | - |  | 385.48 | - | - | - | 385.48 | - | - | - | 108.98 | - | 276.50 | - |
| 13 | - | - | - | - | 213.12 | - | - |  | 213.12 | - | - | - | 213.12 | - | - | - | - | - | 213.12 | - |
| Colorata | - | - | - | - | 204.68 | - | - |  | 204.68 | - | - | - | 204.68 | - | - | - | - | - | 204.68 | - |
| Zanaibar | - | - | - | - | 208.08 | - | - |  | 208.08 | - | - | - | 208.08 | - | - | - | - | - | 208.08 | - |
| 18 | - | - | - | - | 240.49 | - | - |  | 240.49 | - | - | - | 240.49 | - | - | - | - | - | 240.49 | - |
| Bao Luo Lan | - | - | - | - | 436.90 | - | - |  | 436.90 | - | - | - | 436.90 | - | - | - | - | - | 436.90 | - |
| 22 | - | - | - | - | 299.08 | - | - |  | 299.08 | - | - | - | 299.08 | - | - | - | - | - | 299.08 | - |
| 23 | - | - | - | - | 216.30 | - | - |  | 216.30 | - | - | - | 216.30 | - | - | - | - | - | 216.30 | - |
| 27 | - | - | - | - | 423.10 | - | - |  | 423.10 | - | - | - | 423.10 | - | - | - | - | - | 423.10 | - |
| 29 | - | - | - | - | 155.88 | - | - |  | 155.88 | - | - | - | 155.88 | - | - | - | - | - | 155.88 | - |
| 34 | - | **228.56** | - | - | **375.67** | - | - |  | **604.22** | - | 228.56 | - | 375.67 | - | - | - | 327.20 | - | 277.02 | - |
| Fo Shou Lian | - | **89.20** | - | - | **506.15** | - | - |  | **595.35** | - | 89.20 | - | 506.15 | - | - | - | 595.35 | - | - | - |
| Huang Guan Zi | - | **223.18** | - | - | **417.31** | - | 86.96 | 84.39 | **640.49** | 171.34 | 310.14 | - | 417.31 | - | - | - | 362.12 | - | 365.33 | - |
| 42 | - | - | - | - | 176.89 | - | - |  | 176.89 | - | - | - | 176.89 | - | - | - | - | - | 176.89 | - |
| Lan He | - | - | - | - | 88.51 | - | - |  | 88.51 | - | - | - | 88.51 | - | - | - | 88.51 | - | - | - |
| Tai Guo Wang | - | - | - | - | 556.38 | - | - |  | 556.38 | - | - | - | 556.38 | - | - | - | 133.40 | - | 422.97 | - |
| **Amaranth** |  |  |  |  |  |  |  |  |  |  |  |  |  |  |  |  |  |  |  |  |
| Qi Feng | - | 410.03 | 111.36 | - | - | - | - |  | 521.39 | - | 410.03 | 111.36 | - | - | - | - | 115.39 | 111.36 | 294.65 | - |
| 9 | - | 742.05 | 113.55 | - | - | - | - |  | 855.59 | - | 742.05 | 113.55 | - | - | - | - | 139.79 | 113.55 | 602.25 | - |
| 10 | - | 743.39 | 111.56 | - | - | - | - |  | 854.95 | - | 743.39 | 111.56 | - | - | - | - | 138.06 | 111.56 | 605.33 | - |
| Ruby | - | 773.95 | 109.91 | - | - | - | - |  | 883.86 | - | 773.95 | 109.91 | - | - | - | - | 137.84 | 109.91 | 636.11 | - |
| Pink pearl | - | 428.53 | 128.94 | - | - | - | - |  | 557.47 | - | 428.53 | 128.94 | - | - | - | - | 100.07 | 128.94 | 328.46 | - |
| 37 | - | 639.90 | 97.45 | - | - | - | - |  | 737.36 | - | 639.90 | 97.45 | - | - | - | - | 119.89 | 97.45 | 520.02 | - |
| 47 | - | 985.56 | 146.50 | - | - | - | 113.70 |  | **1132.05** | 113.70 | 1099.25 | 146.50 | - | - | - | - | 162.60 | 146.50 | 936.65 | - |
| 48 | - | 630.93 | 90.05 | - | - | - | 81.57 |  | 720.98 | 81.57 | 712.50 | 90.05 | - | - | - | - | 107.49 | 90.05 | 605.01 | - |
| 49 | - | 311.40 | 92.43 | - | - | - | - |  | 403.83 | - | 311.40 | 92.43 | - | - | - | - | 88.99 | 92.43 | 222.41 | - |
| **Red** |  |  |  |  |  |  |  |  |  |  |  |  |  |  |  |  |  |  |  |  |
| Redflagsong | 102.79 | 428.37 | - | 252.98 | - | 141.44 | 191.66 | 114.31 | 784.14 | 447.42 | 620.04 | - | - | 252.98 | 102.79 | 141.44 | 293.56 | - | 467.92 | 252.98 |
| Albert Greenberg | 108.66 | 254.56 | - | - | - | - | - |  | 363.22 | - | 254.56 | - | - | - | 108.66 | - | 254.56 | - | - | - |
| Roxburgh | - | 111.22 | - | 126.36 | 103.01 | - | 966.50 | 112.66 | 340.59 | **1079.16** | 1077.72 | - | 103.01 | 126.36 | - | - | 214.23 | - | 966.50 | 126.36 |
| White |  |  |  |  |  |  |  |  |  |  |  |  |  |  |  |  |  |  |  |  |
| Ai Ji Bai | - | - | - | - | - | - | - |  | - | - | - | - | - | - | - | - | - | - | - | - |
| 20 | - | - | - | - | - | - | - |  | - | - | - | - | - | - | - | - | - | - | - | - |
| He Hua water lily | - | - | - | - | - | - | - |  | - | - | - | - | - | - | - | - | - | - | - | - |
| **Yellow** |  |  |  |  |  |  |  |  |  |  |  |  |  |  |  |  |  |  |  |  |
| Eldorado | - | - | - | - | - | - | - |  | - | - | - | - | - | - | - | - | - | - | - | - |
| Mo Xi Ge Huang | - | - | - | - | - | - | - |  | - | - | - | - | - | - | - | - | - | - | - | - |

^1^ The name of water lily cultivar;

^2^ Dp: delphinidin; Cy: cyanidin; Ga: galactoside; G: glucoside; Rh: rhamnoside; De:derivatives; the content of compounds in μg per 1 g fresh petals. The bold numers were worthy to be attention due to composition of anthocyainin in cultivars

-: not detected.

The unknown compound **a10** only existed in ‘Bao Luo Lan’
